# The in vitro assessment of the toxicity of volatile, oxidisable, redox-cycling compounds: phenols as an example

**DOI:** 10.1007/s00204-021-03036-w

**Published:** 2021-05-25

**Authors:** Laia Tolosa, Teresa Martínez-Sena, Johannes P. Schimming, Erika Moro, Sylvia E. Escher, Bas ter Braak, Bob van der Water, M. A. Miranda, Barbara M. A. van Vugt-Lussenburg, José V. Castell

**Affiliations:** 1 Unidad Mixta de Hepatología Experimental, Instituto de Investigación Sanitaria, Hospital La Fe, Valencia, Spain; 2 Division of Drug Discovery and Safety, Leiden Academic Centre for Drug Research (LACDR), Leiden University, Leiden, The Netherlands; 3 Fraunhofer ITEM, Chemical Safety and Toxicology, Hanover, Germany; 4 BioDetection Systems BV, Amsterdam, The Netherlands; 5 Departamento de Bioquímica y Biología Molecular, Facultad de Medicina, Universidad de Valencia, Valencia, Spain; 6 CIBERHED, ISCIII, Madrid, Spain; 7 Instituto de Tecnología Química, UPV-CSIC, Universidad Politécnica de Valencia, Valencia, Spain

**Keywords:** Toxicity of phenols, Redox-cycling phenols, In vitro toxicity, Hepatotoxicity, Cross-contamination of culture wells, Plastic seals, High-content imaging, HepG2 BAC-GFP SRXN1 assay, Nrf2 CALUX reporter gene assay, Cytotox CALUX reporter gene assay

## Abstract

Phenols are regarded as highly toxic chemicals. Their effects are difficult to study in in vitro systems because of their ambiguous fate (degradation, auto-oxidation and volatility). In the course of in vitro studies of a series of redox-cycling phenols, we found evidences of cross-contamination in several in vitro high-throughput test systems, in particular by trimethylbenzene-1, 4-diol/trimethylhydroquinone (TMHQ) and 2,6-di-tertbutyl-4-ethylphenol (DTBEP), and investigated in detail the physicochemical basis for such phenomenon and how to prevent it. TMHQ has fast degradation kinetics followed by significant diffusion rates of the resulting quinone to adjacent wells, other degradation products being able to air-diffuse as well. DTBEP showed lower degradation kinetics, but a higher diffusion rate. In both cases the in vitro toxicity was underestimated because of a decrease in concentration, in addition to cross-contamination to neighbouring wells. We identified four degradation products for TMHQ and five for DTBEP indicating that the current effects measured on cells are not only attributable to the parent phenolic compound. To overcome these drawbacks, we investigated in detail the physicochemical changes occurring in the course of the incubation and made use of gas-permeable and non-permeable plastic seals to prevent it. Diffusion was greatly prevented by the use of both plastic seals, as revealed by GC–MS analysis. Gas non-permeable plastic seals, reduced to a minimum compounds diffusion as well oxidation and did not affect the biological performance of cultured cells. Hence, no toxicological cross-contamination was observed in neighbouring wells, thus allowing a more reliable in vitro assessment of phenol-induced toxicity.

**Supplementary Information:**

The online version contains supplementary material available at 10.1007/s00204-021-03036-w.

## Introduction

The assessment of the toxicity of chemicals in in vitro assays is frequently hampered by problems related to solubility, volatility or instability. Assessing toxicity of phenolic compounds is of interest because they are widely used by industry, are present in many trade products and as environmental pollutants (Downs and Wills 2020; Kahru et al. 2002; Zapor 2004). Phenols are difficult to handle because of degradation, oxidation (Yamamura 2009) and volatility that might result in artifactual results as well in contamination of neighbouring wells (Piersma et al. 2013). The stability (auto-oxidation) of phenols in in vitro models depends on the chemical structure (number and position of the hydroxyl groups as well as the presence of substituents on the benzene ring) (Passi et al. 1987). The impact of the instability of phenols and potential losses of tested compounds via degradation and/or diffusion has been seldom addressed in in vitro studies (Pradeep et al. 2017; Zhao et al. 2020). Efforts to develop new methods for evaluating the safety of volatile chemicals has been demanded by the new REACH regulation (EUREACH 2020).

As a part of the EU-ToxRisk Project, we set up a read-across exercise to anticipate the toxicity of structurally related phenols. The test set consisted of six hydroquinone like compounds with redox-cycling potential, 12 redox-cycling negative compounds with alkyl side chain and three phenols without an alkyl side chain. The first set of assays used in this read-across case study was a high-throughput battery of CALUX reporter gene assay (Piersma et al. 2013). During testing using the CALUX cells, interferences in neighbouring wells were observed, an issue that led to unexpected inconsistencies in the in vitro results of compounds being assayed at the time, explainable by cross-contamination and losses of assayed compounds. Hence, we have focussed our attention in understanding the phenomena occurring in the course of the in vitro toxicity assessment of phenols and centred our research efforts on the behaviour of two model compounds, namely trimethylbenzene-1,4-diol/trimethylhydroquinone (TMHQ) and 2,6-di-tertbutyl-4-ethylphenol (DTBEP) both representing the two different groups of redox cycling and alkylated compounds.

We examined in detail the behaviour of these compounds in the course of the in vitro assay incubation, by assessing the amount of remaining parent’s compound, its degradation (oxidation) and its diffusion to adjacent wells, by means of Gas Chromatography–Mass Spectrometry (GC–MS) analysis (Lim et al. 2011), as well by monitoring the metabolic effects on nearby cells. An exhaustive analysis revealed that TMHQ displayed a fast degradation kinetic accompanied by a significant diffusion rates through air of some of the oxidation products to neighbouring wells. The stoichiometric balance revealed that the concentration of the compounds in wells decreased, not only by air-diffusion to other wells but also due to oxidation and degradation processes. There was no adsorption or sequestration of parent compounds by the plastic. We could identify several degradation products, formed during incubations. 2,6-di-tertbutyl-4-ethylphenol, on the other hand, showed somewhat lower degradation kinetics and four degradation products could be identified, but still had a high diffusion rate.

This is an artefactual situation, as in both cases in vitro testing underestimated the toxicity of the parent compounds because of a decrease in their starting concentration. In addition, air-diffusion resulted in cross-contamination to other neighbouring wells, interfering in the assay of other molecules in the culture plate (Blein et al. 1991). In a similar contextual problem, it was suggested that the use of impermeable seals could overcome these volatility drawbacks in a fish toxicity study, but never investigated at the physicochemical level (Schug et al. 2020).

To address these limitations, and to make the in vitro study of the toxicity of phenols feasible and reliable, we investigated in detail the physicochemical changes occurring in the course of the incubation and made use of gas-permeable and non-permeable plastic seals that were placed on the top of multi-well plates replacing the conventional plastic lid, to minimize diffusion and oxidation. A non-permeable gas seal was expected to better prevent diffusion and oxidation of phenols but might have drawbacks for the O_2_/CO_2_ exchange and negatively influence cells. Thus, we evaluated as well the effects of the seals on the biological performance of cultured cells.

As a result of this strategy, diffusion and oxidation were greatly diminished; no toxicological cross-contamination was observed in neighbouring wells, and a more reliable, less artefactual in vitro assessment of phenols-induced toxicity was possible.

## Materials and methods

### Reagents

2,6-Di-tert-butyl-4-ethylphenol (DTBEP), CAS Nr. 4130-42-1; Trimethylbenzene-1,4-diol (or trimethylhydroquinone, TMHQ) CAS Nr. 700-13-0 and 4-tert-octylphenol (4-t-OP) CAS Nr. 140-66-9 were purchased from Sigma Aldrich (Madrid, Spain). Stock solutions were prepared by weighing the appropriate amount and dissolving it in DMSO.

Culture media and complements were purchased from GIBCO (Gibco BRL, Paisley, UK). The tested compounds and substrates used for enzyme activity measurements were acquired from Sigma Aldrich (Madrid, Spain). Fluorescent probes CellROX Deep Red was obtained from Molecular Probes, Invitrogen (Madrid, Spain). Propidium iodide (PI), TMRM and Hoechst 33342 were obtained from Sigma Aldrich. Polystyrene 96-well culture plates were obtained from Nunc (Ref. 167008, ThermoScientific, Madrid, Spain).

In order to study the prevention of cross-contamination, two different plastic seals were tested: a polyester non-breathable seal (Platesealer, easyseal transparent, Ref 676001 from Greiner-Bio-one, NC, USA) and a perforated ethylene–vinyl acetate (EVA) gas-permeable film (*QuickSeal Gas Perm* Self Adhesive Film, Ref IST-124-080SS from IST Scientific, UK).

### Culture of HepG2 cells and incubation with test compounds

HepG2 cells (ECACC No. 85011430) were cultured in Ham’s F-12/Leibovitz L-15 (1:1 v/v) supplemented with 7% fetal calf serum, 50 U of penicillin/ml and 50 μg of streptomycin/ml. For subculturing purposes, cells were detached by treatment with 0.25% trypsin/0.02% EDTA at 37 °C. Cells were cultured continuously until passage 20 and were regularly tested for *Mycoplasma* contamination. For the toxicity studies, cells were seeded in 96-well plates (5000 cells/well) and were allowed to grow and equilibrate for 24 h. Then, cells were exposed for 24 h to a range of concentrations (3.9–500 μM) of the test chemicals. Each experimental condition was repeated independently three times (with three wells measured each time). The stock solutions prepared in DMSO were conveniently diluted in the culture medium to obtain the desired final concentrations. The final DMSO concentration in the culture medium never exceeded 0.1% (v/v), and the control cultures were treated with the equivalent amount of solvent.

### HepG2 BAC-GFP SRXN1 assay

HepG2 BAC-GFP SRXN1 cells (Wink et al. 2017) were cultured in Dulbecco’s Modified Eagle Medium high glucose, with 10% (v/v) fetal calf serum, 25 U/ml penicillin, and 25 µg streptomycin. The cells were seeded at a density of 8000 cells per well in 384-well microplate (Greiner bio-one, cat. 781096). Cells were stained for 2 h with Hoechst 33342 at a concentration of 200 ng/ml and subsequently exposed to the phenolic compounds (Schimming et al. 2019) after medium removal under the presence of 100 nm for propidium iodide (PI) and 0.1% (v/v) of dimethyl sulfoxide (DMSO). After 24 h of compound exposure, cells were imaged with a Nikon confocal microscope utilizing imaging channels for Hoechst (excitation 408 nm, filter 450/50), GFP (excitation 488 nm, filter 525/50) and PI (excitation 561 nm, filter 595/50). Images were automatically analysed using CellProfiler (Kamentsky et al. 2011) version 2.1.1 as described previously (Schimming et al. 2019). Fraction of GFP positive cells was determined by setting the times the standard deviation on top of the mean DMSO GFP value as a threshold and every cell above this threshold was considered positive. For the sealed experiments the gas permeable film (*QuickSeal*) was used.

### Nrf2 CALUX^®^ and Cytotox CALUX^®^ reporter gene assays

A stable human U2-OS cell based CALUX^®^ reporter gene assay was used to detect activation of Nrf2 pathway, indicative for oxidative stress. Additionally, the Cytotox CALUX cell line, which detects cell viability, was used. The Nrf2 CALUX and Cytotox CALUX cells were generated and cultured as described previously (van der Linden et al. 2014). Exposure to the test compound TMHQ, dissolved at 0.1 M in DMSO, was performed in 384-well plates for 24 h and at 0.1% DMSO (v/v) according to the assay procedure as described in DM-ALM protocol 197 “Automated CALUX reporter gene assay procedure”. For both assays, the luciferase signal elicited by vehicle control (DMSO) exposed wells was set to 100%, and the signals obtained from the TMHQ-exposed wells and the neighbouring wells were scaled accordingly. The experiment was also performed in the presence of a gas-permeable film (*QuickSeal Gas Perm* Self Adhesive Film, Ref IST-124-080SS from IST Scientific, UK).

### High-content screening assay: incubation with fluorescent probes, imaging analysis

Following treatments, cells were simultaneously loaded with 1.5 µg/ml Hoechst 33342, 1.5 µg/ml PI and 5 µM CellROX. After a 30-min incubation at 37 °C with the culture media containing fluorescent probes, cells were imaged by the INCELL6000 Analyser (GE Healthcare, USA) as previously described (Tolosa et al. 2012).

The cell count was generated from the number of Hoechst 33342-stained nuclei. Cell viability was determined by PI exclusion. Since PI is not permeant to alive cells, this dye is used to monitor detect dead cells in a population. This allows not only the direct quantification of cytotoxicity, but also the exclusion of dead cells from the HCS analysis of other parameters, thus restricting functional determinations to the live-cell population in each sample. Reactive oxygen species (ROS) production was measured as an increase in CellROX fluorescence intensity in the cytoplasm. Each measure was performed in individual cells, the values in the course of the same treatment. For each end-point, triplicate wells were measured and averaged and then normalized by the average value of untreated cells. The captured images were further analysed using the INCELL analysis workstation, which allows the simultaneous quantification of subcellular structures that are stained by different fluorescent probes. The measured fluorescence intensity was associated with the predefined nuclear and cytoplasmic compartments (Tolosa et al. 2012).

### Experimental design for diffusion analytical studies

Two hundred microlitres of either 2,6-di-tert-butyl-4-ethylphenol or trimethylbenzene-1,4-diol/trimethylhydroquinone at 100 μM were added to selected wells in a 96-well plate. Neighbouring wells were filled with 200 μL of culture media and the whole plate covered with the conventional plastic lid or with gas-permeable and non-permeable plastic seals was kept at 37 °C. Two-hundred-microlitre samples of three increasingly distant neighboring wells were obtained after 3, 6 or 24 h in replicated well plates, and extracted with 200 μL of dichloromethane. The original well content was diluted ten times with dichloromethane to fit in the analytical calibration range. The organic phase was stored at − 80 °C until analysis. Calibration solutions were prepared at 10, 5, 2.5, 1, 0.5, 0.25 and 0.1 ppm concentration for each compound.

### Mass spectrometry and chromatographic separation conditions

Analysis was performed on an Agilent 7890B GC system coupled with an Agilent 5977A (Agilent technologies, CA, USA) MS detector. A GC capillary column HP-5msi (30 m, 0.25 mm, 0.25 µM) was used. Helium flow was 1 ml/min. Source temperature was 230 °C, MS quad temperature 150 °C and inlet temperature 250 °C. Two microlitre sample volume was injected on splitless mode. Initial column temperature was 60 °C for 3 min ramped to 280 °C at 20 °C/min and held for 8 min at 300 °C.

### Annotation of peaks and assignation of chemical structures

Peak detection and integration were carried out using Qualitative MassHunter 10.0 software. Assignation of chemical structures was first performed by NIST mass spectra library comparison, as well by knowledge-guided interpretation of mass fragmentation spectra and the chemical reactivity of the compounds.

### Data analysis

Experimental data (usually as triplicates) were evaluated for data quality and expressed as mean ± S.E.M. Statistical significance and *p* values were depicted whenever relevant.

## Results

### In vitro biological cross-contamination occurring when assessing the toxicity and biological activity of phenolic compounds

**Fig. 1:**
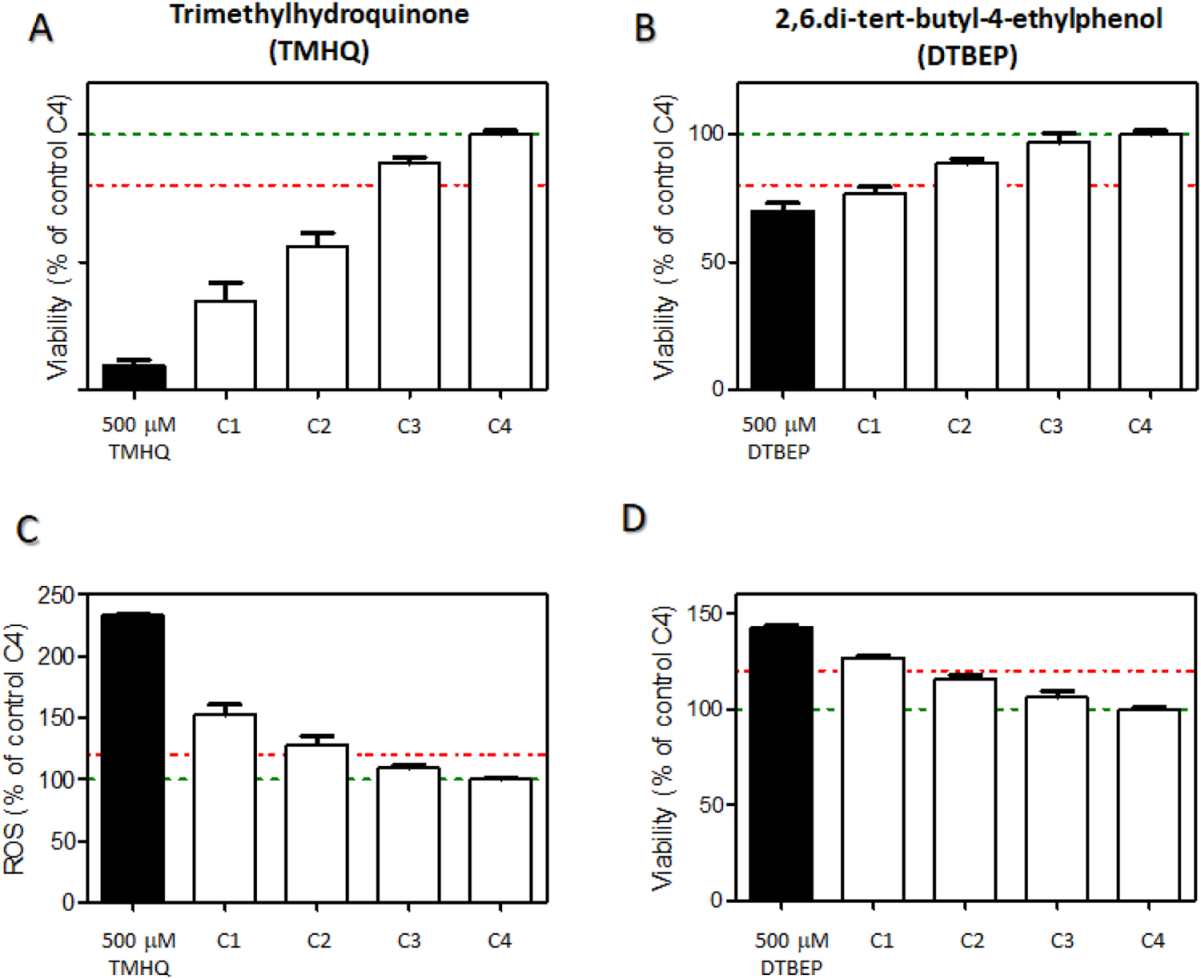
Cross-contamination of phenolic compounds in neighboring wells. HepG2 cells cultured in 96-well plates were incubated with 500 µM trimethylhydroquinone (TMHQ; **a**, **c**), **b** or 2,6 ditertbuthyl-4ethylphenol (DTBEP; **b**, **d**) for 24 h and toxicity was evaluated by means of HCI in treated wells and neighbouring control wells (C1–C4, being C1 the closest well to the treated one). Significant effects in both viability (**a**, **b**) and ROS production (**c**, **d**) were detected not only in treated wells but also in their closest control wells, indicating cross-contamination. Horizontal green dotted line represents the normalized value of non-treated cells; red dotted line represents the threshold of a significant change respect to control cells

In a series of initial in vitro experiments, it was noted that the presence of phenols in wells had a clear distorting influence on the cytotoxicity of other compounds measured in neighbouring wells. To determine the biological magnitude of such effect we monitored its influence on the viability of untreated cells placed at increasingly distant wells. To this end, HepG2 cells were seeded in a 96-well culture plate, with two selected phenolic compounds for 24 h at a given concentration (Fig. 1). The decrease in viability of non-treated cells was more pronounced depending to the proximity to the well containing the phenol (Fig. 1a, b). In a similar manner, the occurrence of reactive oxygen species (ROS) (Fig. 1c, d) depends on the proximity to the treated well, thus evidencing the cross-contamination by the treated well. Control, non-treated cells, situated at 1–4 wells distance (C1–C4) were clearly affected depending on their distance to the starting well.

**Fig. 2:**
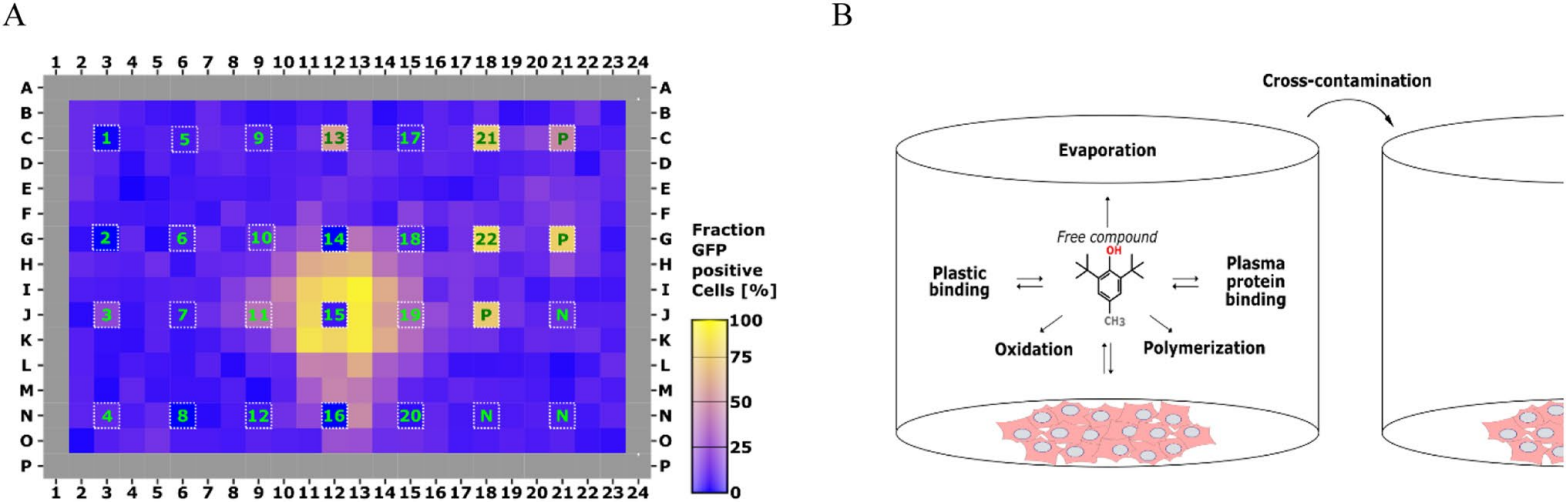
Cross-contamination by phenols as detected by HepG2 BAC-GFP SRXN1 assay. Cross contamination was examined in 384-well microplates covered with a conventional plastic lid. Panel **a** several phenolic compounds (1–22; see Supplementary Table 1), tested at 500 µM and placed at the indicated position; *N* = solvent control 0.1% (v/v) DMSO; *P* = 100 µM diethyl maleate). The surrounding wells were not directly exposed to any compound treatment. Clear effects on neighbouring wells were observed for trimethylbenzenediol, (or TMHQ, 15); Panel **b** conceptual representation of potential fate of phenolic compounds in a microplate test system

Similarly, when other cell reporters were assayed (i.e., SRXN1 or oestrogen receptor activation), an artefactual influence was observed in adjacent wells, as shown in Fig. 2. Especially trimethylhydroquinone (TMHQ) stands out due to the high activity in the HepG2 BAC-GFP SRXN1 assay but also due to a far-reaching cross-contamination into the neighbouring wells (Fig. 2a).

Conceptually, there are several routes such phenolic compounds might interfere in a microplate test system, as depicted in Fig. 2b.

### Losses of assayed compounds, in the course of the in vitro assay

**Fig. 3:**
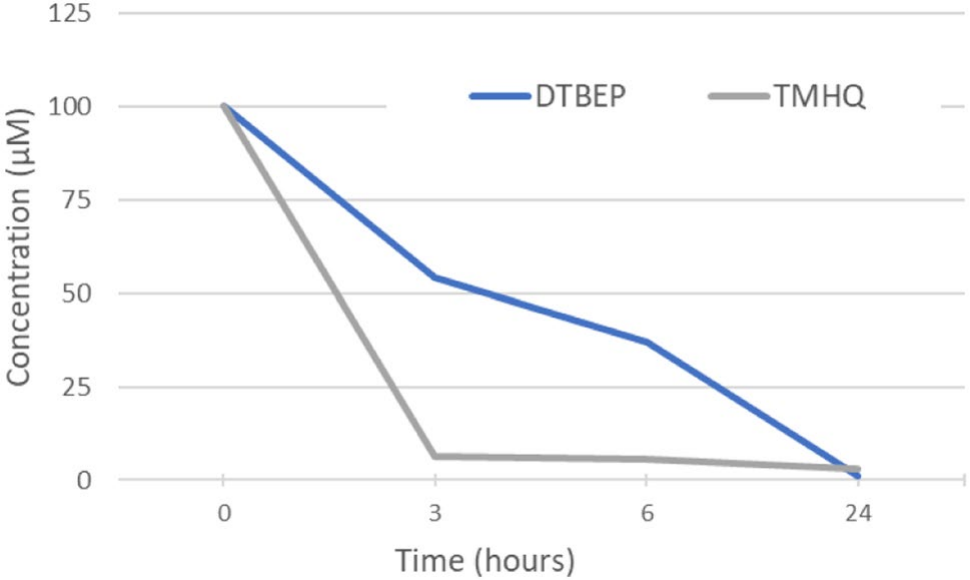
Disappearance of 2,6-di-tert-butyl-4-ethylphenol (DTBEP) and trimethylhydroquinone (TMHQ) in wells covered with conventional plastic lid at 3, 6 and 24 h. Concentrations of both compounds after 3, 6 and 24 of incubation were determined by GC/MS in aliquots of culture media drawn at different times (3, 6, 24 h) and related to the starting (*T* = 0 h) concentration. TMHQ almost totally disappeared just after 3 h of incubation whereas DTBEP remained longer in the well until 24 h

The biological effects so far observed could be due to an “escape” and cross-contamination of wells by the tested compound, in the course of the in vitro assay; a phenomenon that would influence the results of other compounds tested in the same plate (Fig. 2b). To determine the magnitude of the phenomenon, and whether the compound and its concentration remained stable in the original well in the course of the incubation for in vitro testing, we assayed the concentration of the tested compounds along the time of incubation. As depicted in Fig. 3, the concentration of both tested compounds decreased over time, probably as consequence of evaporation/diffusion throughout the multi well plate, contaminating neighbouring wells, or by compound degradation.

### Diffusion of phenols in multi well plates: physicochemical cross-contaminations

**Fig. 4:**
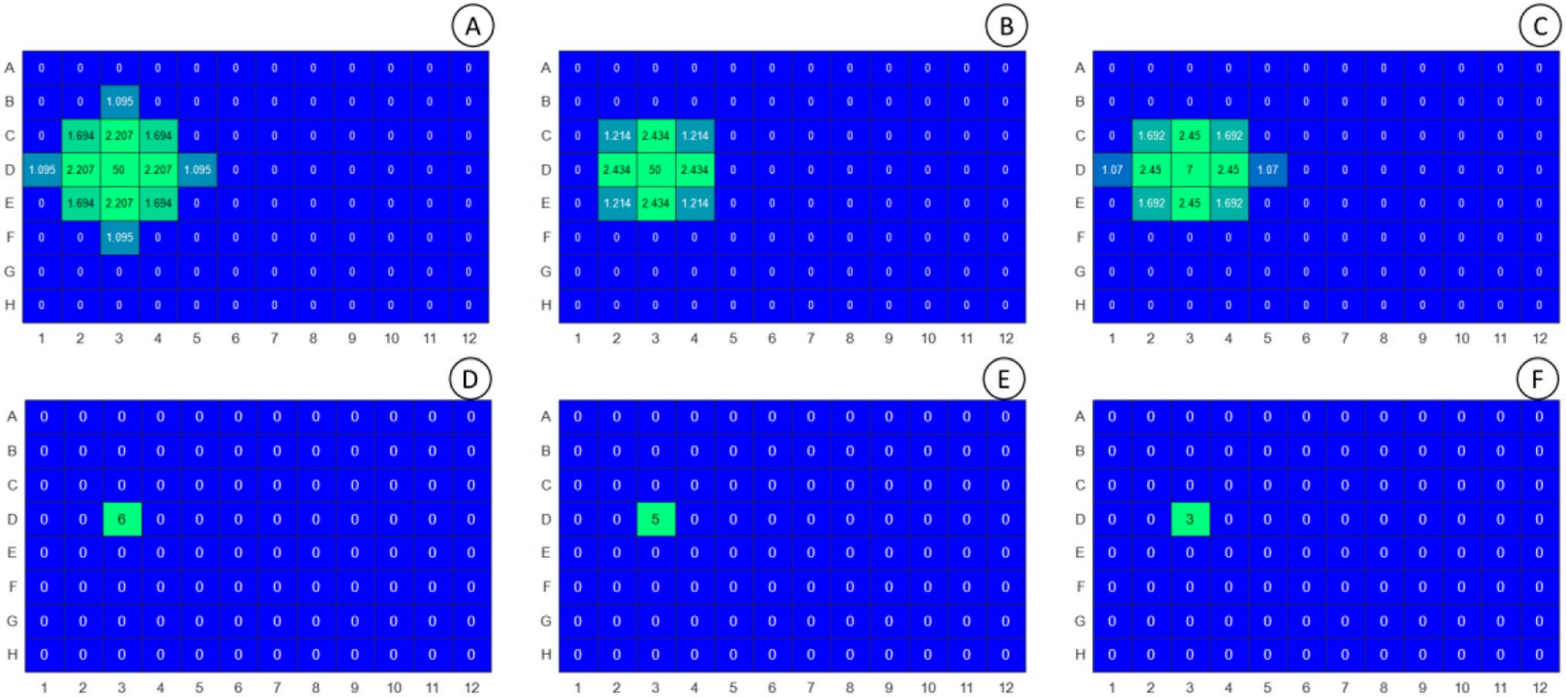
Diffusion of two representative phenols along adjacent wells Diffusion of 2,6-di-tert-butyl-4-ethylphenol at 3, 6 and 24 h (**a**–**c**, respectively) and trimethylhydroquinone (TMHQ) at 3, 6 and 24 h (**d–f**, respectively) in culture plates covered with the conventional plastic lid. Samples of culture media of neighbouring well were taken at 3, 6 and 24 h, extracted with dichloromethane and quantified by GC/MS. Results are depicted as a heat map diagram where intensity of green colour corresponds to the remaining concentration in the wells

The above mentioned results pointed at the diffusion of the phenolic compounds from one well to neighbouring wells, contaminating the culture media of such vicinal wells, as one possible explanation. To assess the extent of the phenomenon, we examined the concentration of the respective phenols in the surrounding wells, after 3, 6 and 24 h of incubation. Samples of culture media of near-by wells were extracted and analysed by GC/MS, searching for the parent molecule. As shown in Fig. 4, there are clear evidences of compounds escaping from the original well, diffusing and re-dissolving in adjacent wells, at least for 2,6-di-tert-butyl-4-ethylphenol. However, we did not detect TMHQ in distant wells, what apparently might exclude its direct role in the cross-contaminating cytotoxicity effects observed in adjacent wells (Fig. 1), but it does not exclude the diffusion of other degradation products from the stating well.

### Mass balance of the incubated phenols

Having found evidences that the phenols cross-contaminated the neighbouring wells, and that this could be related to their ability to escape from their original wells, we calculated mass balance to determine whether the disappearance of the parent compound in the original well explained the amount of compound detected in the contaminated wells. No parent phenols were detected (below the detection limit) in the plastic binding experiment, thus excluding this phenomenon to explain the losses of the compound Thus, as summarised in Table 1, we found a mass imbalance suggesting that on addition to phenol diffusion, degradation (oxidation?) is likely to occur.

**Table 1:** DTBEP and TMHQ mass balance in culture plates after incubation

Compound	Time (h)	Plastic binding (nmol)	Stability (nmol)	Diffusion (nmol)	Possible degradation (nmol)
2, 6-Di-tert-butyl-4-ethylphenol	3	< 0.02	10	3	7
	6	< 0.02	10	3.4	6.6
	24	< 0.02	1.4	3.8	14.8
Trimethylhydroquinone	3	< 0.02	2	< 0.02	18
	6	< 0.02	1	< 0.02	19
	24	< 0.02	1	< 0.02	19

A mass balance was conducted to determine the fate of the phenols. The remaining amount of phenol, plus that detected in the neighbouring wells, as well that attached to plastic, did not explain the losses in the parent compounds at 3, 6 and 24 h. Consequently, degradation of the phenols must take place as well

### Oxidative degradation of phenols in the course of incubation

**Fig. 5:**
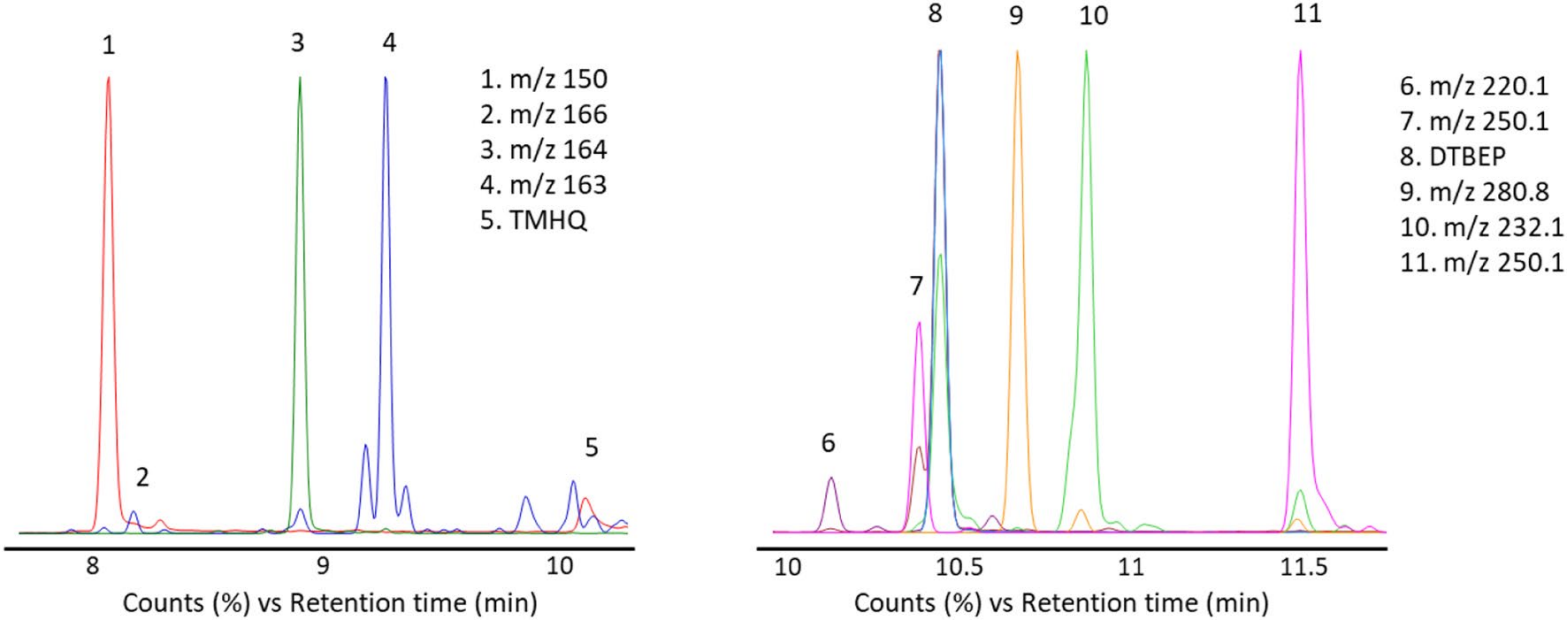
GC/MS analysis of samples extracted from wells containing the assayed phenols. Samples extracted from wells containing the investigated phenols were analysed by GC and detected by means of a coupled mass spectrometer. TMHQ (left) and DTBEP (right) chromatograms of samples after 24-h incubation on culture media, evidenced the occurrence of several peaks

A detailed GC/MS analysis of an extract of the original well containing the phenol, after 24-h incubation, revealed the existence of several compounds eluting at increasing retention times (Rt), for both assayed phenols (Fig. 5). For each individual peak, a full mass spectra were recorded in order to assign a chemical structure (Supplementary Figs. S1, S2, S3).

**Fig. 6:**
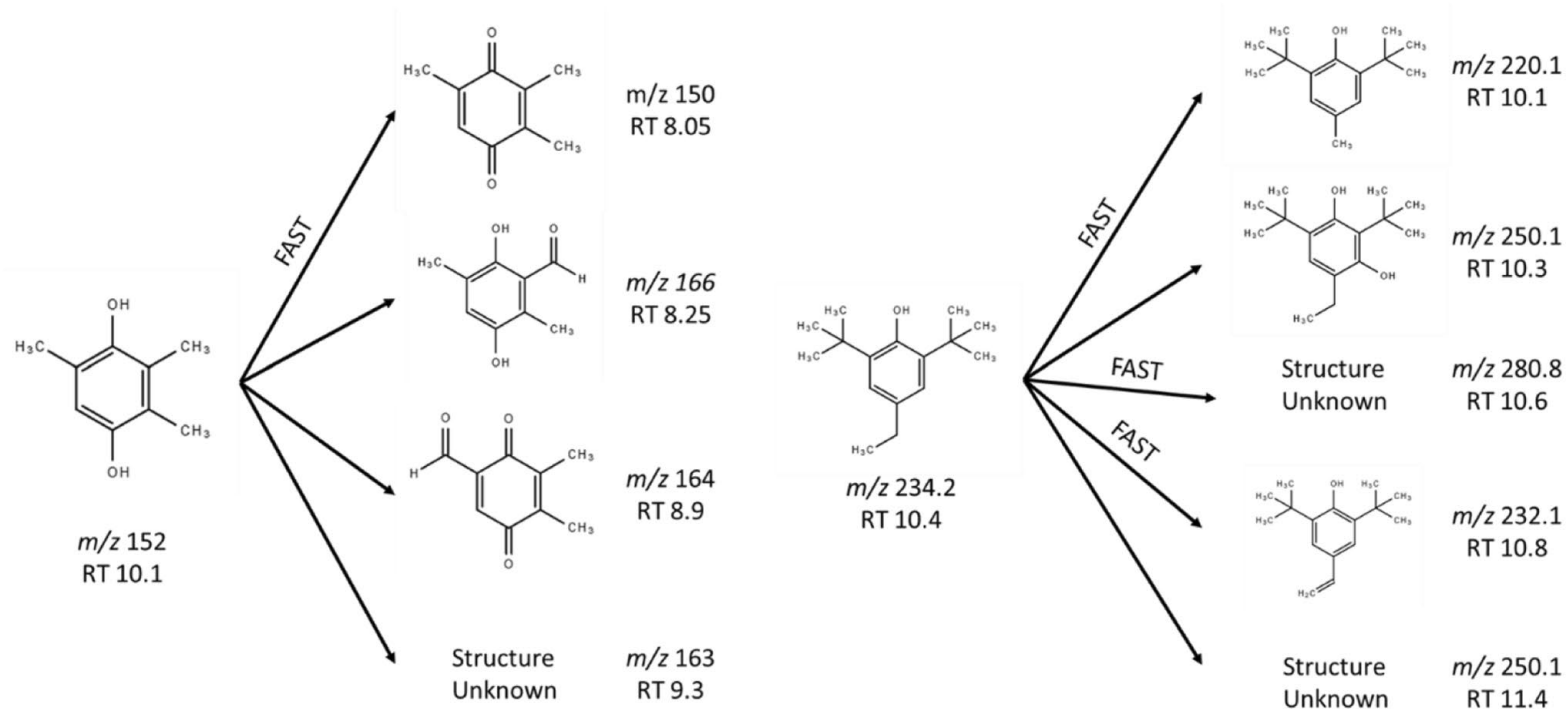
Proposed structures of the degradation products of phenols. The structures of the oxidation compounds of TMHQ (left) and DTBEP (right) were assigned based on their accurate molecular weights and the mass fragmentation pattern. Altogether, the chart displays the feasible oxidation chemical reactions pathways taking place in wells in the course of incubation in plastic lid-covered culture plates

Based on the MS features of parent phenols (Supplementary Fig. S1) and that of the degradation products (Supplementary Figs. S2, S3), we have assigned the structures to the different oxidation products (Fig. 6). TMHQ yielded largely trimethyl quinone (m/z 150—RT 8.05) being the major compound in the solution after 3 h of incubation. Other oxidation derivatives were also structurally identified. Regarding DTBEP, several degradation compounds were found as well, some of them being formed fast in the course of the incubation.

### Air diffusion of oxidised compounds

**Fig. 7:**
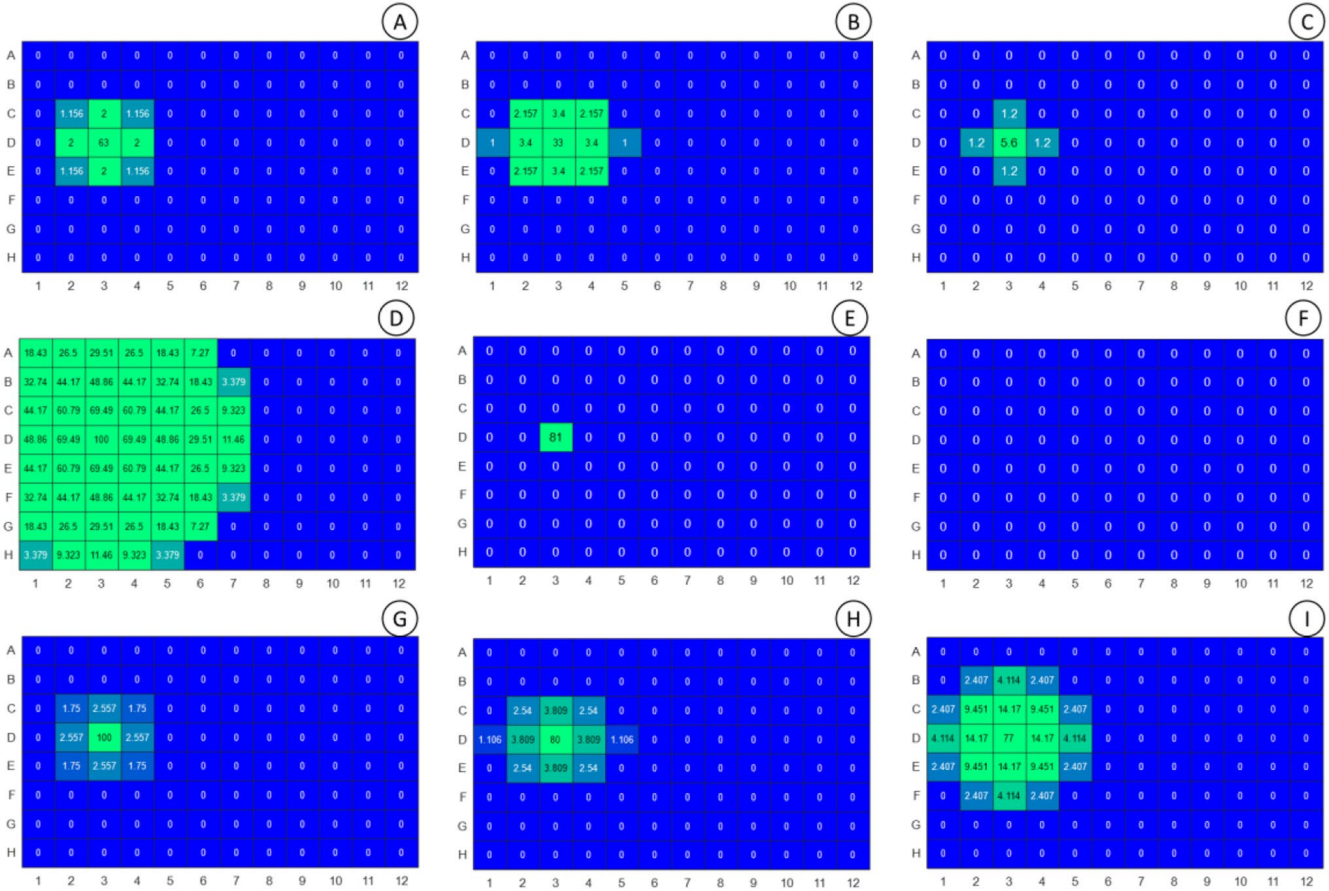
Diffusion of degradation compounds of TMHQ and DTBEP in culture plates covered with conventional plastic lid. **a–c** The diffusion of the quinone of TMHQ at 3, 6 and 24 h of incubation, respectively. The degradation compound of DTBEP with m/z 220.1 and RT 10.1 min (**d** (3 h), **e** (6 h), **f** (24 h)) rapidly diffuses but becomes degraded as well; and the compound m/z 250.1 and RT 10.3 min (**g** (3 h), **h** ( 6 h), **i** (24 h)) was also found at neighbouring wells

When the experiment depicted in Fig. 4 was re-examined, some phenol degradation compounds could also be found at neighbour wells in the incubations covered with conventional plastic lid (Fig. 7). Compound with m/z 150—RT 8.05 min that corresponds to the quinone of TMHQ (m/z 150—T 8.05 min) was examined at 3, 6 and 24 h (a, b and c, respectively) at the percentage values with respect to the sample at time 0 h. The quinone rapidly diffuses but becomes degraded as well. Degradation compounds of DTBEP with m/z 220.1—RT 10.1 min at 3,6 and 24 h (d, e and f) and m/z 250.1—RT 10.3 min at 3,6 and 24 h (g, h, i) are both represented as a percentage of the area of the peak with respect to the time of 3 h since they were not observed at time 0 h.

### Prevention of cross-contamination by the use of plastic seals

**Fig. 8:**
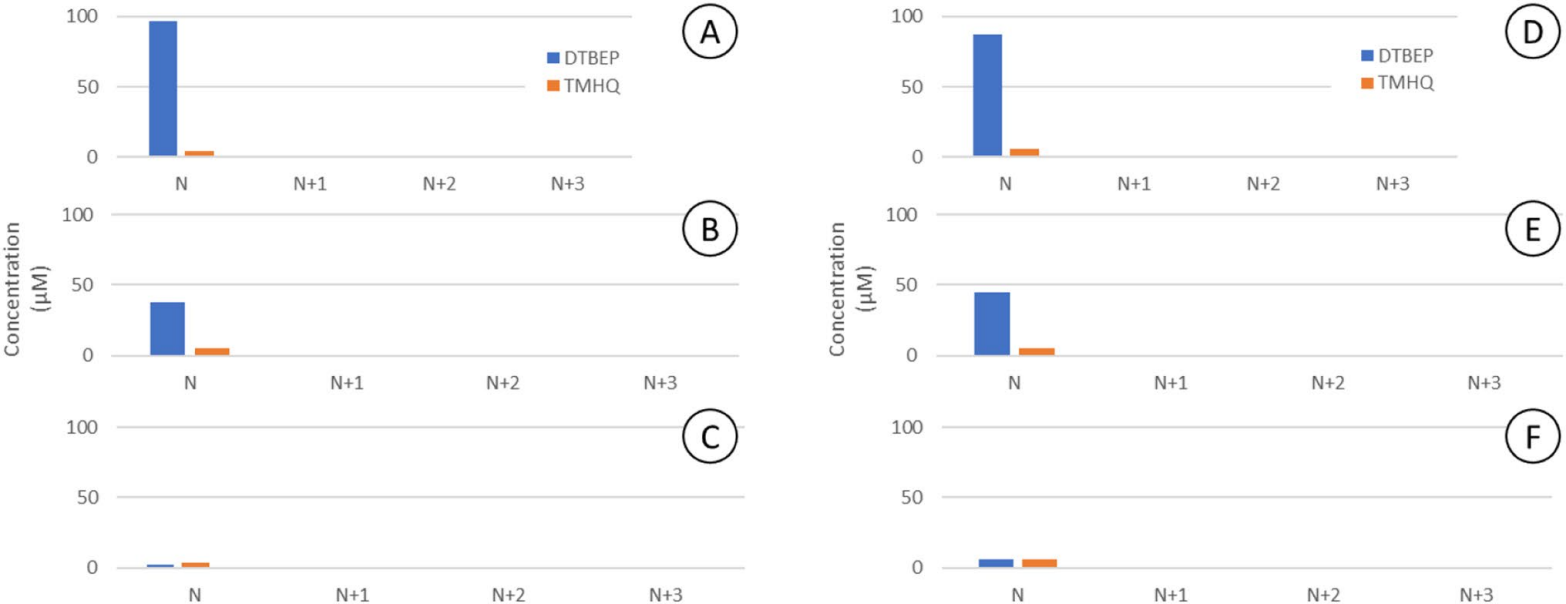
Prevention of cross-contamination by use of plastic seals. Presence of TMHQ and DTBEP in neighbour wells was totally abolished by use of permeable film at 3, 6 and 24 h (**a**, **b**, **c,** respectively), as well with non-permeable film (**d**, **e**, **f** for 3, 6 and 24 h incubation). Seals could not prevent the oxidative degradation of phenols

In order to circumvent the problems associated to cross-contamination and degradation (oxidation) of the tested phenols in the course of the in vitro assays, we covered the multiwell plates with two different types of plastic seals. One is a transparent nanoperforated, gas permeable seal (Gurley 15 s/100 cc/sq) which restricts the diffusion of volatile molecules but allows O_2_ diffusion, whereas the other seal is a polyester adhesive seal that totally prevents gas exchange including O_2_. As depicted in Fig. 8, cross-contamination either by the parent phenols or their oxidation products can be totally prevented with comparable efficiency with both seals. However, they are not fully effective in preventing the oxidation of phenols in the starting wells.

### Plastic seals avoid biological cross-contamination and prevent artefactual results in the in vitro assessment of drug-induced toxicity in HepG2 cells

Since MS analysis demonstrated that plastic seals efficiently avoided diffusion of the tested compounds as well their degradation products, the next step was to demonstrate that whether it also worked for in vitro toxicity experiments in HepG2 cells, and whether the plastic seals had any negative influence per se on cells.

**Fig. 9:**
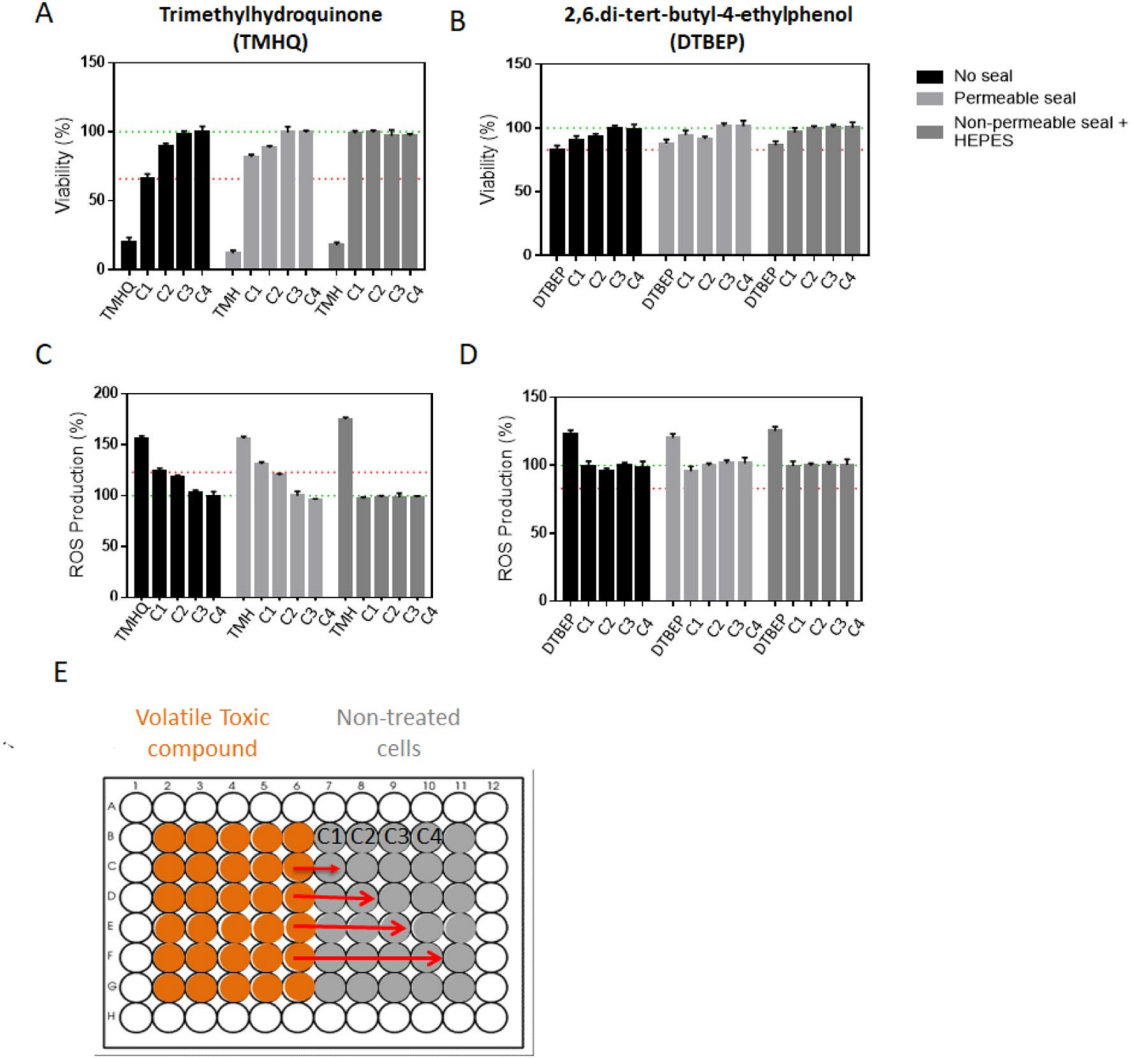
Biological effects of the use of plastic seals on cultured cells. Viability (**a**, **b**) and ROS production (**c**, **d**) was determined by means of high-content screening in HepG2 cells exposed to 500 µM of trimethyl hydroquinone (**a**, **c**) or 2,6-ditert-butyl-4ethylphenol (**b**, **d**) and control cells (C1–C4) located in neighbouring wells. Cross-contamination was clearly prevented when a non-permeable seal was used and partially prevented with the permeable seal. **e** Schematic representation of the culture plate and the distribution of non-treated wells (C1–C4)

When the permeable or the non-permeable seals were used and the toxicity of trimethyl hydroquinone and 2,6-di-tert-butyl-4-ethylphenol was assessed in the starting and in neighbouring, control, i.e., non-treated cells, it became evident that the formerly observed cross-contamination could be fully avoided as the precedent GC–MS analysis data revealed. Although no big differences between permeable and non-permeable seal were observed, it seemed that the non-permeable seal is somewhat better avoiding cross-contamination of TMHQ since a clear cut off was observed both in viability and ROS production among treated and non-treated cells (Fig. 9).

The Cytotox CALUX assay, performed in 384-well plates, showed that 100 µM TMHQ caused almost complete cell death (Fig. 5S, top). In the adjacent wells, C1–C4, however, the cell viability was 93% (C1) to 100% (C3–C4), indicating that the gas-permeable seal prevented cross-contamination to a large extent. For the Nrf2 CALUX assay (oxidative stress, figure X bottom), a similar trend was observed. Exposure to 10 µM TMHQ resulted in significant Nrf2 activation (430% of blank). In the neighbouring well C1, 160% activation was observed, indicating that limited cross-contamination occurred, but in wells C2–C4 no Nrf2 activation was detected. In conclusion, in the case of TMHQ exposure of CALUX cells in 384-well plates, the cross-contamination could be largely prevented using a gas-permeable seal.

**Fig. 10:**
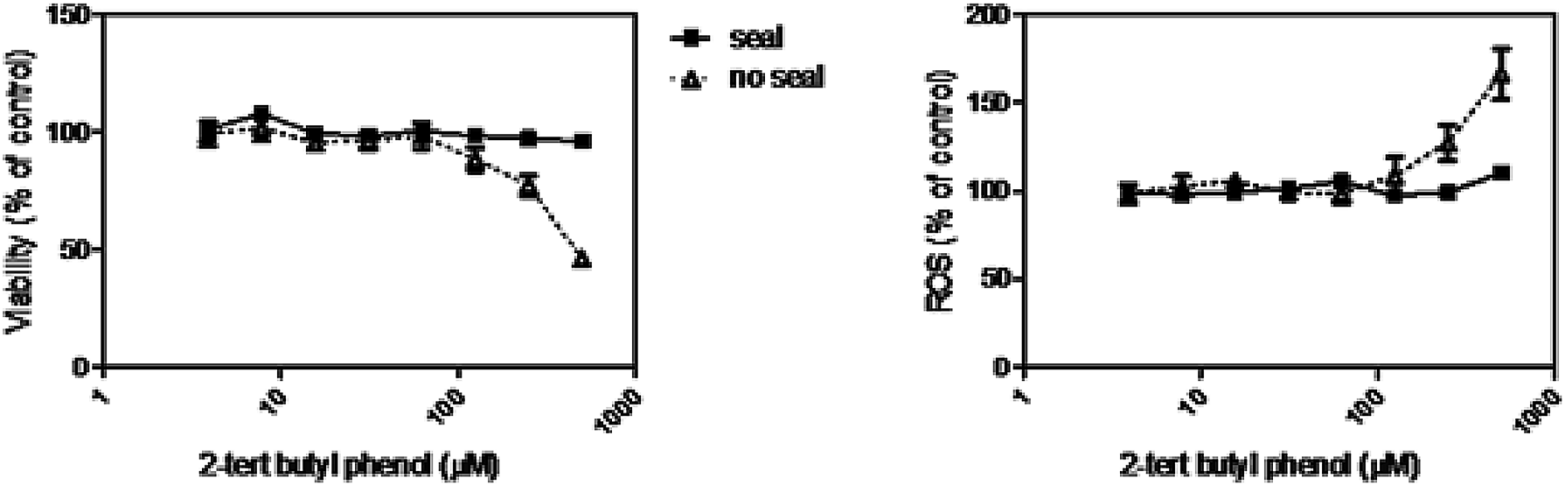
Avoidance of toxicological cross-contamination and artefactual results when using non-permeable plastic seal. For some of the compounds it has been evidenced that their volatility could affect the neighbour wells (control cells or treated with other compounds), affecting the results and interpretation. The experiment of the figure displays the toxicity of 2-tert-butylphenol when in the same plate a volatile compound, trimethylhydroquinone, was present and how the use of a seal prevented the occurrence of artifactual results

Finally, to demonstrate the importance of minimizing cross-contamination in the assessment of cytotoxicity, the toxicity of a non-volatile compound (2-tert butyl phenol), poorly cytotoxic, was assessed in the same plate, close to wells containing trimethyl hydroquinone, and the effect of the plastic seal was comparatively studied. Different cytotoxicity curves and effects on ROS production were obtained in the absence or presence of the seal, evidencing a clear of cross-contamination effect and confirming the importance of the seals in preventing such an artefact in the study of the toxicity of volatile compounds (Fig. 10).

Finally, another important issue demanded attention, i.e., to which extent the use of plastic seals altered the normal performance of cultured cells. To this end we examined viability, ROS production and mitochondrial membrane potential of non-treated cells either under conventional culture conditions (plastic lid), or sealed with membranes. No significant differences were observed in any of the recorded parameters when compared to non-treated cells cultured with plastic lid (without seal) and cells with the two distinct types of seals (permeable and non-permeable), during the incubation times evaluated (Supplementary Fig. S5).

## Discussion

In vitro assessment of toxicological properties of compounds under conventional submerse assay conditions may lead to misleading potency values in case that tested compounds are (semi) volatile or yield volatile oxidisable/degradable products. Besides that, the tested compounds may air-diffuse from the originally applied well, and interfere with testing of other compounds by cross-contaminating neighbouring wells. A second source of uncertainty is the degradation or oxidation of test compounds during the incubation media. We have been confronted by this situation in the course of the in vitro toxicity evaluation of redox-cycling phenols. In the present paper, different physico-chemical mechanisms were thoroughly investigated, quantifying the occurrence of degradation and diffusion of volatile phenols/or of their degradation products along the incubation in vitro, and the conditions and clues to circumvent this problem when assessing compounds of this nature, have been also been defined.

Initially, the in vitro assays in this study were planned to support the hazard characterization of a group of compounds in a read-across setting. Read-across is a well-sustained strategy for predicting endpoint information (toxicity) for one substance (target substance), by using data from the same endpoint from other similar substances. It is becoming quite useful for safety assessment of not-yet in vivo assayed compounds, and makes use of in vitro and in silico data, facilitating the estimation of human hazards (Escher et al. 2019). In the EU-ToxRisk project, a read-across case study on alkylated phenols and hydroquinones, was designed to explore in how far in vitro models can be used to characterize the hazard of these compounds and to support the justification of different subcategories. To enable the comparison of in vitro derived potency values, the in vitro conditions will have to assure that compounds are tested under standardised and comparable conditions. Within the selection process of the grouped compounds, physico-chemical parameters were used to estimate the most appropriate testing conditions. The molecular weights and vapour pressure values of the investigated phenols did not directly alert for volatility, e.g. trimethylhydroquinone (TMHQ) has a molecular weight of 152 Da and a vapour pressure of 0.03 Pa, while 2,6-di-tert-butyl-4-ethylphenol (DTBEP) had a MW of 234 Da and a VP of 0.3 Pa. Nevertheless, during in vitro testing, cross-contamination of neighbouring wells was observed, an issue that strongly interfered in the experimental outcomes. Despite the presumable lower diffusion ability of TMHQ, yet its detection in neighbouring wells, is explained by the rapid oxidation that will affect to any diffused TMHQ molecule, generating trimethyl quinone, which is the active compound in the SRXN1 reporter assay.

The use of plastic seals prevented air diffusion. Indeed, they dramatically reduced the cross-contamination of vicinal wells and only, in part, the oxidative degradation of phenols. Gas non-permeable seals were somewhat more effective than the semi-permeable seals but in spite of this could not prevent the auto oxidation of phenols. Oxygen dissolves in aqueous media to reach a concentration close to 200 µM at 37 °C (Al-Ani et al. 2018), a sufficient amount to oxidise phenols that are tested at a similar or lower concentration range. Aromatic compounds such as phenols and their derivatives can indeed undergo oxidation by molecular oxygen present in the air or dissolved in the aqueous media. These reactions have been extensively studied (Vogel et al. 1999) giving rise to oxidation compounds and dimerization. The disappearance of the parent compounds and the formation of oxidation mixtures were evident in the course of the incubation; however, we found no evidences of a significant formation of dimers or multimers (polyphenols) in the assay conditions.

Plastic absorption was not responsible for the decrease of the initial concentrations of the assayed compounds, rather the degradation by oxidation accounted for the major part of the decrease in concentration of phenols. Altogether, results evidenced that air diffusion and oxidation are relevant phenomena that cannot be ignored, occurring with the parent phenol, as well with some of the oxidation derivatives, and it is the principal cause of toxicological cross-contamination of vicinal wells, caused by the parent compound or by any of its oxidation derivatives.

The quantitative use of in vitro toxicity assays to determine safe exposure levels in humans requires extrapolation from the media concentrations that produce a significant effect to the in vivo exposures that would be expected to produce an effect (Campbell et al. 2015). Before quantitative in vitro to vivo extrapolations (QIVIVE) are safely implemented, standardization methods that take into account uncertainties of the assessment of volatile/oxidizable compounds are needed. Our study has not only revealed that cross-contamination uncertainties may be avoided by using a non-permeable seal, but also points out that volatility and instability of the parent compounds should be seriously considered in the in vitro assays. If this learning is not undertaken the uncertainty for ranking compounds relative to each other will be high. Unfortunately, this is seldom addressed within literature in scientific reports on in vitro tests of alkylated phenols/hydroquinones. A certain degree of uncertainty may remain when comparing in vitro data to the in vivo situation, where oxidation/degradation kinetics might differ. Quantitative in vitro to in vivo extrapolation QIVIVE, an essential element for in vitro-based risk assessment, will have a higher uncertainty, but assays with the strategies here indicated will remain suitable for hazard identification.

The tested concentrations were high to visualize the phenomena; nevertheless, in vitro testing is commonly performed at the maximal concentration of the compound in blood (*C*_max_) multiplied by a factor that usually ranges between 20 and 100 (O’Brien et al. 2006). For predictive hazard estimations, it should be considered that although HepG2 cells are widely used for hepatotoxicity studies, their principal drawback is lack of biotransformation enzymes, particularly P450 enzymes, what implies they are unable to detect metabolism-mediated hepatotoxicity (Donato et al. 2008). This, however, can be easily circumvented by the use of upgraded adenovirus-transfected HepG2 cells transiently expressing high and functional levels of the five major human CYPs (Tolosa et al. 2013).

By conducting in vitro experiments under these premises, toxicological cross contamination could be greatly avoided and the quality and consistency of results increased by diminishing the artefactual contribution of diffusion and degradation of the assayed compounds. The coverage of culture wells with plastic seals did not negatively influence the biological functionality of cultured target cells. Altogether, these results open the possibility to meaningfully optimize the experimental design and performance of the in vitro experiments, whenever the toxicity of volatile and oxidisable compounds are to be assayed. However, in addition to the prevention of cross contamination by using seals, attention to the potential volatility and instability of this kind of compounds should be paid in order to improve the interpretation and quality of the results. Despite the uncertainties of the in vitro assessing of volatile compounds compared to the in vivo situation, the proposed strategy and deep analysis of degradation and cross-contamination should enable proper hazard identification.

In summary, diffusion was greatly prevented by the use of both plastic seals, as revealed by GC–MS analysis of the content of the neighbouring wells. Gas non-permeable plastic seals, reduced to a minimum diffusion as well oxidation. When experiments were run with cells cultured under these conditions, cell functionality in control wells was not affected by the plastic seals and no toxicological cross-contamination was observed in neighbouring wells.

## Supplementary Information

Below is the link to the electronic supplementary material.

Supplementary file1 (DOCX 349 kb)

Supplementary file2 (DOCX 13 kb)

## Data Availability

The datasets generated during and/or analysed during the current study are available from the corresponding author on reasonable request, including the metadata, and the curated and annotated peak table as MATLAB.mat files.
