# Supplementary material for: The in vitro assessment of the toxicity of volatile, oxidisable, redox-cycling compounds: phenols as an example
Source: Arch Toxicol. 2021 May 25;95(6):2109–21. doi: 10.1007/s00204-021-03036-w (PMC8166692; doi:10.1007/s00204-021-03036-w)
Supplement: Supplementary file 1 — Supplementary file1 (DOCX 349 kb) [file 204_2021_3036_MOESM1_ESM.docx]

**SUPPLEMENTARY FIGURES**


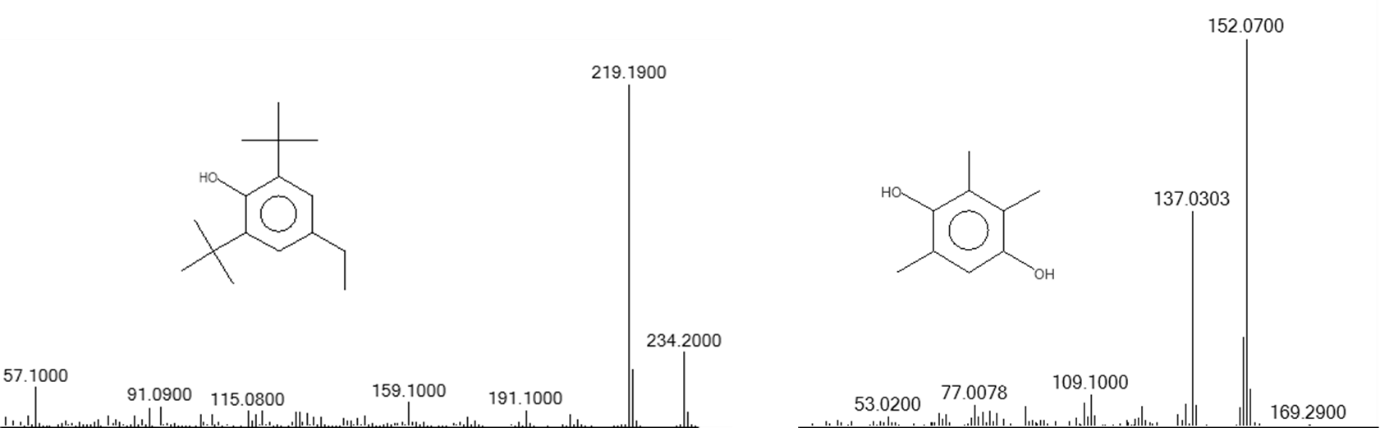


***Supplementary Figure S1.*** Mass spectra of 2,6-di-tert-butyl-4-ethylphenol (left) and trimethylhydroquinone (right).


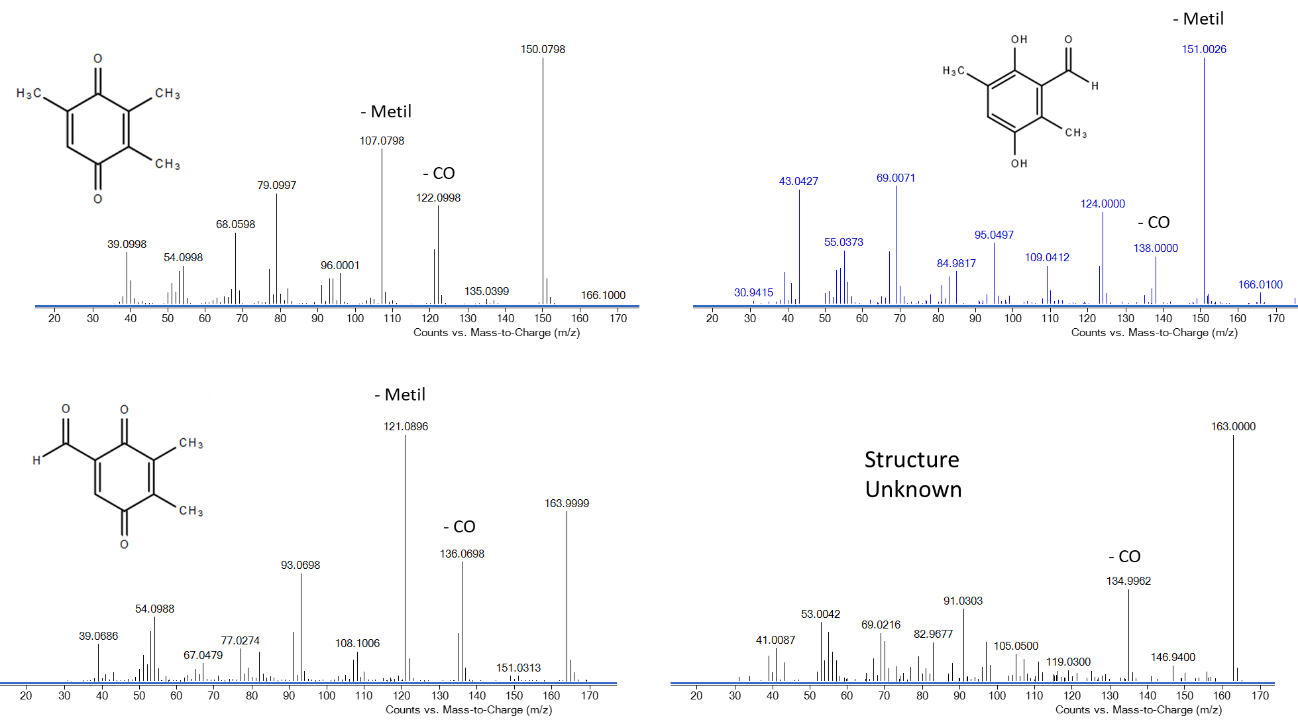


**Supplementary Figure S2**. Mass spectra of the degradation compounds spectra of TMHQ.


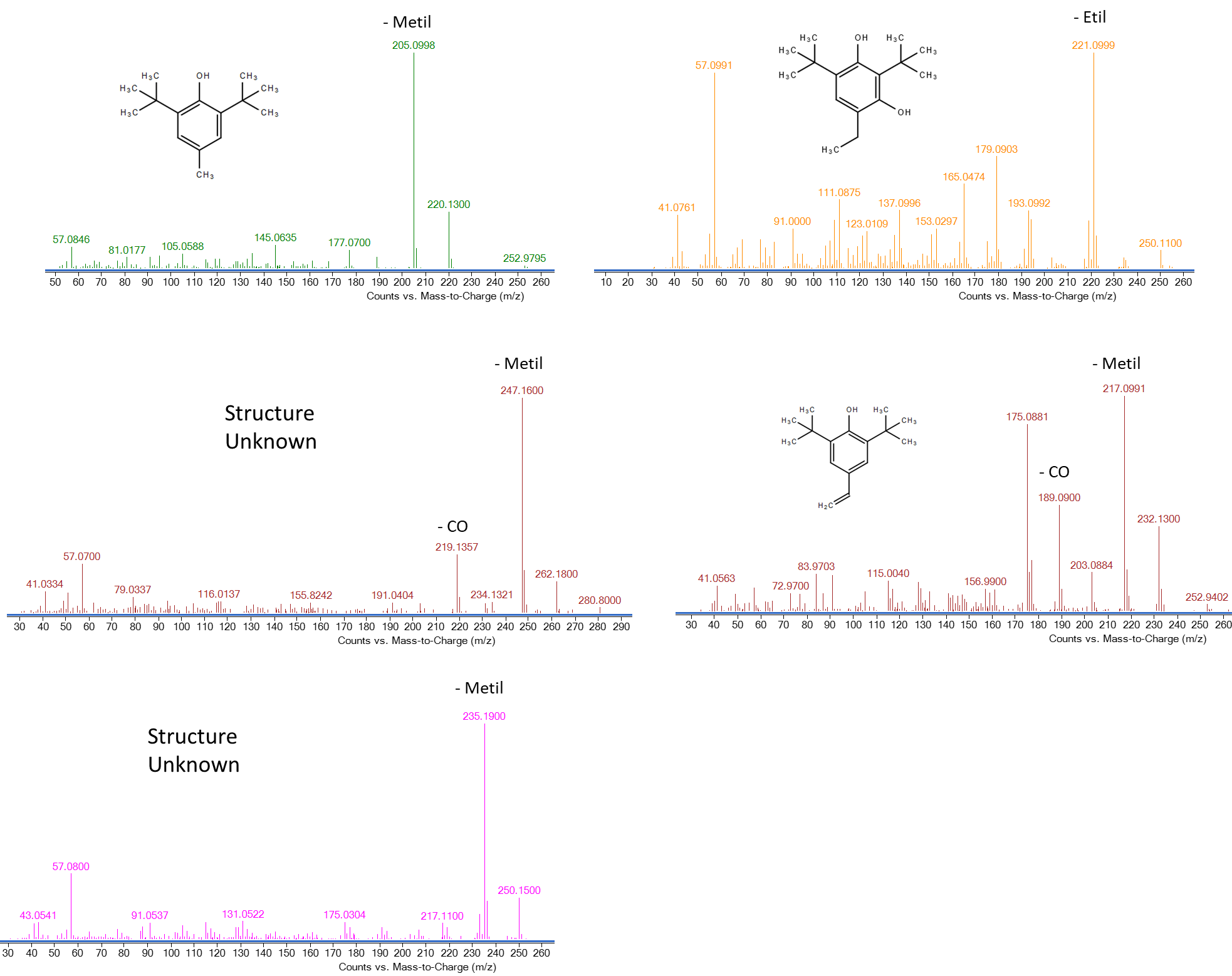


**Supplementary Figure S3**. Mass spectra of the degradation compounds spectra of DTBEP.


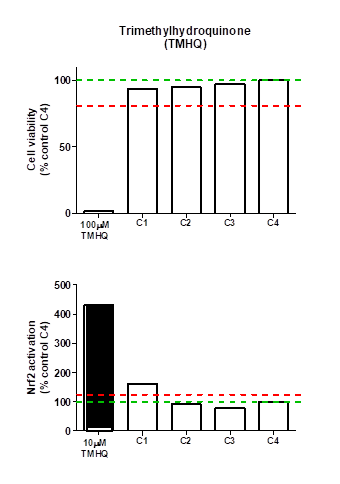


**Supplementary Figure S4. Cross-contamination detected by the Nfr2 CALUX assay.** Results are plotted as Nfr2 activation in % compared to the control C4 well. The results show that with the use of the plastic seal interferences even in close vicinal wells are minimised. Horizontal green dotted line represents the normalized value of non-treated cells; red dotted line represents the threshold of a significant change respect to control cells


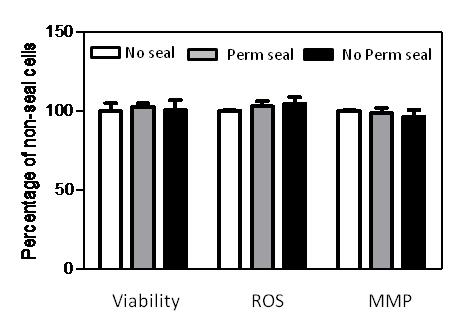


**Supplementary Figure S5. Effects of seals on cell integrity (viability, ROS production and membrane mitochondrial potential) of control, non-treated cells.** The viability, basal ROS production and mitochondrial membrane potential (MMP) were assessed by HCS in HepG2 cells after 24h in the presence or absence of the plastic seal. No significant differences were observed between the control cells (covered with conventional with plastic lid) or sealed with permeable or non-permeable seal.
