# Supplementary material for: The in vitro assessment of the toxicity of volatile, oxidisable, redox-cycling compounds: phenols as an example
Source: Arch Toxicol. 2021 May 25;95(6):2109–21. doi: 10.1007/s00204-021-03036-w (PMC8166692; doi:10.1007/s00204-021-03036-w)
Supplement: Supplementary file 2 — Supplementary file2 (DOCX 13 kb) [file 204_2021_3036_MOESM2_ESM.docx]

**Supplementary table 1:** List of compounds.

| Compound ID | Name | CAS |  |  |
| --- | --- | --- | --- | --- |
| 1 | 2-(1,1-dimethylethyl)phenol | 88-18-6 |  |  |
| 2 | 2,4-Di-tert-butylphenol | 96-76-4 |  |  |
| 3 | p-tert-Butylphenol | 98-54-4 |  |  |
| 4 | 2,4-Dimethylphenol | 105-67-9 |  |  |
| 5 | 4-Methylphenol | 106-44-5 |  |  |
| 6 | 2,6-Di-tert-butyl-p-cresol | 128-37-0 |  |  |
| 7 | 2,6-Di-tert-butylphenol | 128-39-2 |  |  |
| 8 | 4-*tert*-Octylphenol | 140-66-9 |  |  |
| 9 | 2,6-Dimethylphenol | 576-26-1 |  |  |
| 10 | 2,3,5-Trimethylphenol | 697-82-5 |  |  |
| 11 | 2,3,6-Trimethylphenol | 2416-94-6 |  |  |
| 12 | 2,6-Di-tert-butyl-4-ethylphenol | 4130-42-1 |  |  |
| 13 | Catechol | 120-80-9 |  |  |
| 14 | Hydroquinone | 123-31-9 |  |  |
| 15 | Trimethylbenzene-1,4-diol/ Trimethyl hydroquinone | 700-13-0 |  |  |
| 16 | ter-Butylhydroquinone | 1948-33-0 |  |  |
| 17 | Resorcinol | 108-46-3 |  |  |
| 18 | 1,3,5-Benzenetriol | 108-73-6 |  |  |
| 19 | Phenol | 108-95-2 |  |  |
| 20 | Diquat bromide monohydrate | 6385-62-2 |  |  |
| 21 | 2-Methyl-1,4-naphthoquinone | 58-27-5 |  |  |
| 22 | N,N,N',N'-Tetramethyl-p-phenylenediamine dihydrochloride | 637-01-4 |  |  |
